# Celluloid Splinting from Plaster Casts

**Published:** 1919-09-20

**Authors:** 


### PLASTER WORK.

### II. Celluloid Splinting from Plaster Casts.

When quiescence of tuberculous bone or joint trouble has been secured by the means already described, it has to be maintained. For this purpose some sort of splint is advisable, if possible, made so as to be removable at night. Obviously the better the fit, the more efficient, and the more practicable and comfortable, the splint will be. Both these qualities are secured by celluloid (non-inflammable) splinting constructed from a plaster cast of the part to which it is to be applied. Celluloid is very light, too; a hip splint of this material is far lighter than a Thomas' splint. It is also a far better fit. At present it loses a little by comparison in the matter of cost, but with the re-establishment of peace conditions things may alter in that respect. It takes more time and trouble to construct, but that, in view of the better result obtained, is matterless.

### The Making of the Cast.

The first step is the making of the cast. To do this we put on a hip spica already described; but with three differences: (*a*) no stockinette combinations, (*b*) the skin is oiled with olive oil to prevent the plaster sticking thereto, (*c*) very much less bandage is used, only a few turns round the thigh and body to make a framework, and hot instead of cold water to facilitate speedy setting. Then the plaster is cut off almost at once, long before it is hard, by a longitudinal median incision down abdomen and thigh, the line of this incision having previously lines drawn at right-angles to it with a blue pencil to facilitate accurate reposition of the plaster when removed. This last is carefully done and the plaster put away in a corner to harden. When hard, the two apertures for the buttock and thigh below are closed by a few turns of a plaster bandage. When this too has set, plaster of a semi-liquid consistency is poured in from above (cheaper plaster will do for this, of a pinkish tint). To prevent the addition from adhering to the interior, that is previously daubed over with soft soap. When the new plaster has set solid and hard, the old casing is removed, and you have the cast.

The cast is to be covered with *two* thicknesses of stockinette, and to make these fit well a little of the dressmaker's craft is necessary, small cuts being made and pleats taken up. When a smooth and tight fit is obtained, book muslin soaked in celluloid solution has to be applied layer by layer. The celluloid (scrap celluloid is cheapest, and it has to be of low nitrification) is dissolved in acetone. During the war only methyl acetone was obtainable, which has a disagreeable smell, but now the better kind may be procured. The technique of dissolving the celluloid is important. Take one quart of celluloid and cover with the same quantity of acetone, or rather with as much as will give it the consistency of thin cream. The quantity of the acetone varies, because in hot weather it evaporates more quickly than in cold. Add (to secure non-inflammability) three ounces (Gauvain) of granulated calcium chloride dissolved in two ounces of boiling water, added gradually, to every gallon of acetone plus celluloid of proper consistence aforesaid. Then use this to paste on strips about eighteen inches wide of book muslin, snipping them to make them fit well in to the waist. Strengthen the sides with extra slips. About twenty layers are needed, book muslin being thin, and only two coats are possible in a day, or in hot weather, when drying is quicker, three or four. The process can be hastened by using the thicker stockinette instead of book muslin, fewer layers being then needed, but with the present war prices, this raises the cost. The last coat is given three coats of acetone, the final one of which is well polished by hand before quite dry. (N.B.—Some hands get dermatitis and need rubber gloves). You may say that the biggest kind, an average sized spinal jacket, takes a gallon of acetone and costs about £2.

### Separating the Splint.

Now the cast is clothed with the splint as with a skin, and that skin has to be removed. With a bootmaker's knife cut it down the mid-line in front. Here comes the rationale of the two thicknesses of stockinette on the cast. One of them is necessarily sacrificed, and the other is left as a smooth lining to the splint. Two subsequent processes finish the job. The edges are bound, the two bordering the median incision with leather furnished with eyelet holes, through which a lace is put with which to fasten the splint on. A cobbler will do this. Lastly, the splint is strengthened by quarter-inch strips of duralumin, secured to it with copper rivets. The length of the above process obviously makes it advisable for several splints to be in hand at the same time. The Alton instructions to patients are that splints should be well scrubbed, both outside and in, with monkey soap and warm water at least once a week; that the skin should be kept well washed and lightly powdered; and that a thin vest should be worn under the splint.

This type of splinting is particularly permanent and lasting, and, as suggested, may be readily cleaned without in any way destroying its strength or rigidity. To keep it in good condition in the instance of a quiescent non-discharging tuberculous focus is relatively simple, but, although the description has been given primarily for this type of lesion, it must be remembered that there is great utility for this method in the later stages of compound fractures or even old-standing tubercle where slight discharge may occur from wounds. The celluloid structure has considerable advantages of permanency and strength over the paraffin bandage type, and the yet older plaster and dextrine structures invariably become offensive by absorbing the discharges from such cases. The celluloid method can definitely claim, in the long run, cheapness, strength, lightness, cleanliness, and an extremely neat appearance.

